# Endometriosis During Peri-Menopause and Post-Menopause: A Review of the Literature

**DOI:** 10.3390/jcm14228067

**Published:** 2025-11-14

**Authors:** Mayumi Raheem, George Condous, Mercedes Espada Vaquero

**Affiliations:** Department of Obstetrics and Gynaecology, Nepean Hospital, Kingswood, NSW 2747, Australia

**Keywords:** endometriosis, menopause, diagnosis of endometriosis, malignant transformation, endometriosis-associated ovarian cancer

## Abstract

Endometriosis is traditionally regarded as a condition predominantly affecting women of reproductive age, often associated with infertility and cyclical pelvic pain. As a result, a significant body of research and clinical attention has been directed toward the younger patient population. However, there is growing recognition that endometriosis can persist or even arise anew in peri-menopausal and post-menopausal women, yet the impact of the disease in this group remains underappreciated. Many women may have lived with undiagnosed or misdiagnosed endometriosis for decades, often being reassured that period pain and pelvic discomfort were normal aspects of menstruation, and therefore not subjected to appropriate investigation or intervention. This review aims to highlight the clinical significance of endometriosis in peri-menopausal and post-menopausal women. We will examine the common symptoms encountered in this population, discuss current strategies and challenges in diagnosis, and review evidence-based approaches to management. Special consideration will be given to the complex interface between endometriosis and HRT, as well as the potential risk of malignant transformation. Finally, drawing from existing guidelines and expert opinion, we propose recommendations for the diagnosis, treatment, and long-term follow-up of these patients, with the goal of improving outcomes and quality of life for this often overlooked cohort of women.

## 1. Introduction

Endometriosis is traditionally regarded as a condition predominantly affecting women of reproductive age, often associated with infertility and cyclical pelvic pain. As a result, clinical and research efforts have largely focused on younger women. However, increasing evidence shows that endometriosis can persist or even develop in peri- and post-menopausal women—a group that remains under-recognized [1].

Contrary to the historical assumption that endometriosis resolves after menopause due to declining estrogen levels, emerging evidence suggests that residual disease can remain active and symptomatic. In some cases, symptoms may even worsen, particularly in the context of hormone replacement therapy (HRT) or other sources of endogenous or exogenous estrogen [2]. Moreover, the post-menopausal presentation of endometriosis may differ from the classical manifestations seen in women who menstruate, leading to diagnostic delays and missed opportunities for early intervention [3].

This review explores the clinical relevance of endometriosis in peri- and post-menopausal women. It discusses common symptoms, diagnostic challenges, and current management strategies, with particular focus on the role of HRT and potential malignant transformation. Finally, it offers evidence-based recommendations to improve care and outcomes for this often-overlooked group of women.

## 2. Epidemiology and Pathology in Older Women

The true prevalence of endometriosis in peri-menopausal and post-menopausal women is difficult to ascertain, largely due to underdiagnosis, misdiagnosis, and the historical focus on younger populations. Retrospective studies have reported incidental findings of endometriosis in 2–5% of post-menopausal women undergoing surgery for unrelated gynecological indications, although the true burden may be higher given the asymptomatic or atypical nature of the disease in this age group [4,5].

Epidemiological data are further limited by systemic gaps that limit access to care. Prevalence estimates may be especially inaccurate in low-resource settings and among non-White populations, where access to care is limited, reproductive symptoms may carry stigma, and healthcare disparities persist. Older women may also normalize symptoms such as pelvic pain or gastrointestinal discomfort, while clinicians may overlook them, contributing to ongoing delays in diagnosis [1]. Moreover, research has historically centered on White cohorts, reducing the applicability of findings to more diverse populations highlighting the need for inclusive culturally sensitive studies.

### 2.1. Persistence or Recurrence After Menopause

It was once assumed that menopause, through the natural decline in estrogen, would lead to the regression of endometriosis. However, clinical evidence now shows that the disease can persist—and in some cases become more symptomatic—after menopause [6]. This continued activity is believed to be driven by multiple factors, including both internal (endogenous) and external (exogenous) sources of estrogen [2].

### 2.2. Biological Mechanisms Sustaining Persistent or De Novo Disease

Emerging insights highlight several drivers of ongoing pathophysiology in older women. Research shows that progesterone resistance, immune cell dysfunction and epigenetic reprogramming as well as de novo lesions are theories that cause the persistence of endometriosis in menopausal women.

Progesterone resistance is a hallmark of endometriotic tissue and plays a critical role in the persistence and chronicity of disease, though its role in post-menopausal women is less well established, given the physiological decrease in progesterone after menopause. However, one must consider the history of progesterone resistance in women with endometriosis after menopause, especially when hormone replacement therapy (HRT) is considered a treatment strategy when surgical management is contraindicated due to other comorbidities [7]. Endometriotic lesions frequently show reduced expression of progesterone receptor (PR) isoforms, particularly PR-B, which reduces the endometrium’s normal response to progesterone’s anti-inflammatory and anti-proliferative effects. This dysregulation results in a failure to suppress local estrogen activity and inflammatory cytokine production, thereby promoting lesion survival [8]. Clinically, this progesterone resistance may limit the effectiveness of progestin-based therapies in some women during peri-menopause and post-menopause and has implications for HRT choices in this population cohort of patients with endometriosis, who may require individualized strategies that bypass or mitigate progesterone-resistance pathways.

Immune cell dysfunction and epigenetic reprogramming are increasingly recognized as key drivers of persistent inflammation in endometriosis, even in the hypoestrogenic state of menopause. Endometriotic lesions exhibit an altered immune microenvironment, with recruitment and activation of immune cells—particularly macrophages. These macrophages are often skewed toward a macrophage 2 (M2)-like phenotype, which paradoxically promotes tissue remodeling, angiogenesis, and chronic inflammation rather than resolving it. They secrete elevated levels of pro-inflammatory cytokines such as interleukin (IL-1β, IL-6), and tumor necrosis factor α, (TNF-α), which maintain a permissive environment for lesion survival and nerve sensitization [9]. Compounding this, epigenetic alterations can downregulate PRs and contribute to progesterone resistance, further undermining immune homeostasis. These processes may persist independently of systemic estrogen levels, explaining the continued activity of endometriotic lesions in post-menopausal women or those with surgically induced menopause. Together, these findings support a model of endometriosis as a chronic inflammatory disease associated with immune-epigenetic dysregulation, rather than a hormonally dependent condition alone—emphasizing the need for therapies that target immune modulation and epigenetic repair in addition to hormonal suppression.

### 2.3. Role of Residual Estrogen

Even after menopause, low levels of circulating estrogen remain, primarily due to the peripheral aromatization of androgens in adipose tissue [9]. Obesity, therefore, can contribute to sustained estrogen production and has been proposed as a risk factor for the persistence or reactivation of endometriosis in older women [3]. Additionally, local estrogen production by endometriotic lesions themselves, mediated by upregulated aromatase activity, may sustain lesion viability and inflammatory activity independently of systemic hormone levels [9].

### 2.4. Iatrogenic Factors

Exogenous estrogen exposure through HRT is another recognized contributor to post-menopausal endometriosis activity. While HRT is often necessary to manage the vasomotor and genitourinary symptoms of menopause, unopposed estrogen therapy, in particular, has been associated with reactivation of previously quiescent disease and, in rare cases, malignant transformation [10,11]. Gemmell et al. (2017) performed a systematic review discussing the role of unopposed estrogen HRT being associated with the recurrence or activation of endometriosis in women post-hysterectomy and/or bilateral salpingo-oophorectomy [12]. Several case series described the recurrence of pelvic pain or masses attributed to reactivated endometriosis after starting estrogen-only therapy. Similarly, tamoxifen (a selective estrogen receptor modulator commonly used in breast cancer treatment) has partial agonist effects on endometrial tissue and has been implicated in the stimulation of endometriotic lesions [6]. Morgan and Gincherman (1994) reported on a post-menopausal woman receiving tamoxifen for breast cancer who developed a large pelvic mass later confirmed to be endometriosis on histology [6].

## 3. Clinical Presentation in Peri-/Post-Menopausal Women

The clinical presentation of endometriosis in peri-menopausal and post-menopausal women often differs from that in younger women, contributing to significant diagnostic delays. Unlike in reproductive-aged patients, where symptoms are typically cyclical and closely tied to menstruation, peri-menopausal and post-menopausal women may present with non-specific or non-cyclical symptoms, often mimicking other pelvic or gastrointestinal pathologies [3].

### 3.1. Non-Cyclical Pelvic Pain

Chronic pelvic pain remains a common complaint among women with endometriosis during peri-menopause and post-menopause. The pain may be persistent or intermittent, often lacking the predictable pattern associated with menstrual cycles. This makes it harder for both patients and clinicians to recognize endometriosis as the underlying cause. Pain may result from ongoing inflammatory activity, fibrosis, or nerve involvement, and can be severe enough to impact quality of life [1].

### 3.2. Bowel and Bladder Symptoms

Endometriosis involving the bowel and bladder can lead to a variety of gastrointestinal and urinary symptoms. These include dyschezia, constipation, diarrhea, tenesmus, hematuria, urinary urgency, and urinary increased frequency. Because such symptoms are often attributed to common age-related conditions such as irritable bowel syndrome, diverticular disease, or urinary tract infections, the diagnosis of endometriosis may not be considered unless imaging or surgical exploration is performed [6]. In some cases, extrinsic compression or direct invasion of the urinary tract (particularly of the ureters) by endometriotic lesions can cause obstructive symptoms requiring surgical intervention [10].

### 3.3. Pelvic Mass or Post-Menopausal Bleeding

In women during peri-menopause or post-menopause, a newly identified pelvic mass or unexpected vaginal bleeding typically raises concerns about malignancy, often prompting expedited imaging, tumor marker evaluation, and surgical exploration. However, in rare cases, endometriosis can be the underlying etiology. Ovarian endometriomas, while less common during peri-menopause and post-menopause, may persist and undergo morphological changes such as hemorrhage, fibrosis, or complex cystic degeneration, mimicking features suspicious for ovarian neoplasm on ultrasound or MRI [1,3]. Case reports describe peri-menopausal and post-menopausal women presenting with adnexal masses initially presumed to be malignant but ultimately diagnosed as benign ovarian endometriotic cysts at histology [3,13]. In some instances, vaginal bleeding during post-menopause has been attributed to endometriotic foci involving the uterus, cervix, or vaginal vault, either through direct infiltration or secondary to hormonal stimulation from exogenous estrogen exposure [2].

### 3.4. Atypical Presentations and Extra-Pelvic Disease

The lack of classic cyclical symptoms, combined with the overlap between endometriosis-related symptoms and those of other common peri- and post-menopausal conditions, frequently leads to delayed or missed diagnosis. Many women report longstanding pelvic pain or bowel/bladder dysfunction that was either normalized or misattributed earlier in life, only coming to clinical attention during investigations for unrelated complaints in later years. Awareness of the potential for endometriosis to persist or present in this age group is therefore critical for timely diagnosis and management [9].

Extra-pelvic endometriosis can persist or newly manifest in peri-menopausal and post-menopausal women, often at sites less hormonally dependent such as the bowels, urinary tract, diaphragm, lungs, and skin. Case reports have described diaphragmatic and pleural endometriosis presenting as recurrent pneumothorax after menopause, sometimes exacerbated by HRT [10,14]. Similarly, urinary tract endometriosis has been identified in peri-menopausal and post-menopausal women, with ureteric obstruction or bladder lesions requiring surgical management [5,15]. Persistent colorectal endometriosis has also been reported, occasionally presenting with rectal bleeding [4]. Rarely, abdominal wall scar endometriosis may persist or be diagnosed many years after cesarean deliveries [11]. These cases highlight the need for continued clinical suspicion, even in the hypoestrogenic environment of peri-menopause and post-menopause, when evaluating atypical thoracic, urinary, gastrointestinal, or cutaneous symptoms.

## 4. Diagnosis: Imaging and Biomarkers

In line with ESHRE 2022 guidelines, a two-step diagnostic pathway is recommended: initial trans-vaginal or trans-rectal ultrasound (TVUS or TRUS) and magnetic resonance imaging (MRI) if needed—by specialized operators, followed by hormonal therapy in symptomatic patients not pursuing fertility [14]. Diagnostic laparoscopy with histological confirmation remains necessary when imaging is inconclusive or medical treatment with hormonal suppression fails, particularly to detect superficial endometriosis (SE) or rule out malignancy. Patients with suspected deep endometriosis (DE) particularly when there is extra-gynecological or extra-pelvic involvement, should be referred to multidisciplinary specialist centers. Caution must be used when interpreting these pathways, as the ESHRE guidelines are currently not specifically designed for peri-menopausal and post-menopausal women with endometriosis.

### 4.1. Role of Transvaginal Ultrasound and Magnetic Resonance Imaging

TVUS and TRUS remain the first-line imaging modality for evaluating pelvic pain or masses in women of any age. In peri- and post-menopausal women, TVUS and TRUS can identify persistent ovarian endometriomas (OE) typically appearing as unilocular cysts with homogeneous low-level internal echoes (“ground-glass” appearance) [14,16,17]. However, aging-related changes in pelvic anatomy and the potential for coexisting pathology, such as fibroids or adnexal neoplasms, can complicate interpretation.

MRI offers soft tissue characterization and is particularly useful when TVUS or TRUS findings are inconclusive. It can help to distinguish OE from other adnexal masses and assess for extra-pelvic involvement, which may be more common in older women [18]. On MRI, OE characteristically exhibit high signal intensity on T1-weighted images with shading on T2-weighted sequences, although atypical appearances can occur, especially in chronic or hemorrhagic lesions. TVUS is reported to have sensitivity of 93% and specificity of 96% for the diagnosis of OE. While MRI has a comparable performance, it has been shown to be more useful in the mapping of non-ovarian disease [19]. Despite their value, neither TVUS, TRUS or MRI is definitive for excluding ovarian malignancy, and suspicious ultrasound features (e.g., papillary projections, solid components, ascites) should prompt a referral to a gynecology–oncology multidisciplinary team and surgical evaluation [20].

### 4.2. Limitations of CA-125 and Non-Invasive Biomarkers

CA-125 is often elevated in endometriosis but is neither sensitive nor specific, particularly in peri- and post-menopausal women. Levels may be influenced by a range of benign and malignant conditions, including ovarian cancer, pelvic inflammatory disease, and even heart failure [20]. In peri- and post-menopausal women, the use of CA-125 to distinguish OE from ovarian malignancies is especially limited, as malignancy rates increase with age and benign disease often produces only mild elevations of CA-125.

Human epididymis protein 4 (HE4) has emerged as a promising non-invasive biomarker for distinguishing benign endometriotic lesions from malignant transformation, particularly endometriosis-associated ovarian cancer (EAOC). Unlike CA-125, which is often elevated in both benign and malignant gynecological conditions, HE4 tends to remain within normal ranges in benign endometriosis, thus offering greater specificity for malignancy. The combination of HE4, CA-125 and CEA (carcinoembryonic antigen) can improve sensitivity and specificity when evaluating ovarian masses. However, current evidence remains insufficient to recommend HE4 as a routine diagnostic tool for endometriosis or to predict malignant transformation in peri- and post-menopausal women. Further research is necessary to validate its clinical application in this subgroup [20].

A 2016 Cochrane review by Nisenblat, Bossuyt, Farquhar, Johnson, and Hull investigated combinations of clinical, biochemical, and imaging tests [21]. Although promising combinations such as IL-6 + PGP 9.5 for pelvic endometriosis and vaginal exam + TVUS for rectovaginal disease achieved high accuracy in isolated studies, methodological limitations and lack of validation preclude their clinical adoption. Thus, laparoscopy remains the diagnostic gold standard and combined non-invasive tests currently remain within the realm of investigative research.

Gupta et al. (2016) published a Cochrane review looking at endometrial biomarkers (e.g., Pro-Gly-Pro (PGP) 9.5, B-cell lymphoma (BCL)-6, integrin β3) in the diagnosis of endometriosis and found that none achieved adequate sensitivity or specificity to replace diagnostic laparoscopy [22]. Studies were of low methodological quality and heterogeneous, precluding reliable meta-analysis or clinical application. The authors concluded that endometrial biomarkers remain investigational, and laparoscopy remains the definitive diagnostic standard [22].

Recent advancements in non-invasive diagnostics for endometriosis include the use of salivary microRNA, which are conserved, non-coding RNAs involved in gene regulation [23]. The ENDO-miRNA study analyzed saliva samples from 200 women with suspected endometriosis and found that 76.5% of those with a diagnostic miRNA signature were confirmed to have the disease. Ongoing multi-center trials using next-generation sequencing and artificial intelligence have reported promising results, with a sensitivity of 96.2% and specificity of 95.1%. These biomarkers are not yet recommended for use in post-menopausal women [24].

## 5. Treatment: Indications for Surgery and HRT

When managing endometriosis during peri-menopause and post-menopause, there are several factors to consider. While malignant transformation is always a concern in this cohort of women (particularly in the post-menopausal population), one must not discredit the increased cardiovascular risk in women with endometriosis in general, regardless of age [25]. A population-based cohort study of over 160,000 women in Ontario, Canada found that endometriosis was associated with a modestly increased risk of cardiovascular events (adjusted HR 1.14–1.30) compared with matched controls. The increased risk was observed for both hospital admissions and emergency visits, regardless of diagnosis method [26]. This highlights the importance of managing cardiovascular risk factors while considering HRT and surgery.

Surgery plays an important role in the primary treatment of endometriosis—whether due to recurrence of pain in the context of endometriotic lesions, suspicious OE, cystic ruptures or non-gynecological symptoms such as hematochezia with a known bowel lesion or hematuria with a known ureteric or bladder lesion. Additionally, given the increased risk of malignancy in peri- and post-menopausal women, surgical management is often the primary treatment strategy for endometriosis in this population. Initial detection via transabdominal ultrasound (TAUS) should be followed by TVUS/TRUS and MRI to assess the extent of disease, identify DE lesions, and assist in surgical planning. If imaging suggests malignancy, referral to a gynecologic–oncology multidisciplinary team is warranted for appropriate staging and treatment [27].

Medical management may be an option for patients with benign imaging who are not good surgical candidates or who experience pain recurrence after surgery [7,27]. In symptomatic women receiving HRT management, options include increasing the progestin-to-estrogen ratio, discontinuing estrogen, or switching from tamoxifen to aromatase inhibitors (AIs), which help suppress extra-ovarian estrogen production, the primary source of endogenous estrogen in post-menopausal women [26].

In five published post-menopausal cases treated with AIs, doses of 1–5 mg/day for 4–15 months led to symptom relief and lesion size reduction; in one detailed case, 9-month anastrozole therapy resulted in nearly complete lesion regression, though lumbar spine bone mineral density (BMD) fell ~6.2% [28,29]. Thus, while AIs show great promise in the management of symptom recurrence for women with endometriosis during peri- and post-menopause, consideration must be given to the significant side effect profile of this class of drug.

According to the British Menopause Society, add-back hormone therapy in pre-menopausal women who undergo surgically induced menopause is typically administered at doses sufficient to preserve BMD yet low enough to minimize the risk of endometriosis reactivation. The management of menopausal symptoms in this group should be individualized, considering symptom severity and cardiovascular risk profiles. While there is limited high-quality evidence on the rates of endometriosis recurrence or malignant transformation following bilateral oophorectomy, such events have been reported in the literature. A Cochrane review by Al Kadri et al. in 2009 evaluated the effects of HRT in women with a history of endometriosis who entered surgical menopause (typically following oophorectomy, with or without hysterectomy) [30]. The authors searched multiple databases up to March 2008 for randomized controlled trials comparing HRT versus no treatment in this population. Only two small randomized controlled trials met inclusion criteria: one compared transdermal 17β-estradiol + cyclic medroxyprogesterone acetate vs. nonstop tibolone, and the other compared sequential estradiol-progestogen patches + oral progesterone vs. no treatment [31,32].

Encouragingly, a recent study demonstrated no increased risk of malignant transformation over six years of follow-up in women treated with either estrogen-only or combined HRT regimens [25]. Expert consensus suggests that in women who have undergone oophorectomy and have minimal or no residual disease, HRT does not appear to confer a significant additional risk of recurrence or ovarian malignancy. However, in cases where residual disease is substantial, HRT may still be appropriate, particularly in women younger than 45 or those experiencing severe menopausal symptoms, provided that the benefits outweigh the potential risks [7]. While no consensus exits to recommend a specific form of HRT in post-menopausal women with endometriosis, low quality evidence gives different modes of HRT to consider when managing a post-menopausal woman with a history of endometriosis (Table 1).

### 5.1. Malignant Potential and Risk of Transformation

While endometriosis is a benign condition, it has been recognized as a precursor lesion for certain types of ovarian cancer, particularly clear cell and endometrioid carcinomas. This association is particularly relevant in the peri-menopausal and post-menopausal population, where the risk of malignant transformation may be heightened by prolonged estrogen exposure, residual disease activity, or iatrogenic factors such as hormone replacement therapy (HRT) [11,36].

### 5.2. Endometriosis-Associated Ovarian Cancer (EAOC)

Ovarian cancer only develops in 0.3–1.6% of women with endometriosis [37]. Endometriosis-associated ovarian cancer (EAOC) accounts for approximately 10% of all epithelial ovarian cancers. The malignancies most associated with endometriosis are ovarian clear cell carcinoma and endometrioid carcinoma, both of which have distinct clinical and molecular features compared to high-grade serous carcinoma [36,37,38,39,40,41,42]. Studies have demonstrated that atypical endometriosis—characterized by cytologic atypia or architectural complexity—often coexists adjacent to these malignancies, suggesting a continuum from benign endometriosis to cancer development [43,44]. A recently published study looking at endometriosis topology and ovarian cancer risk in a Utah-based cohort of over 500 women, showed that compared with women without endometriosis, the ovarian cancer risk in women with endometriosis was 4.2 times higher. In this population, individuals with both DE and OE had a 9.66 times higher risk of developing ovarian cancer compared to individuals without endometriosis [37].

Patients with EAOC tend to present at an earlier stage and have better survival outcomes than those with high-grade serous carcinoma. Nonetheless, the diagnosis can be challenging, particularly when an OE displays suspicious features on ultrasound such as mural nodules, papillary projections, or rapid growth on imaging [39,40,41,42,43].

### 5.3. Atypical Endometriosis and Clear Cell/Endometrioid Carcinoma

Histologically, atypical endometriosis is defined by the presence of cytological atypia (nuclear pleomorphism, hyperchromasia, increased mitotic activity) or architectural abnormalities such as papillary or cribriform patterns. Atypical endometriosis is found more frequently adjacent to ovarian clear cell and endometrioid carcinomas than in cases of benign endometriosis alone [43]. Molecular studies have identified shared genetic mutations between endometriotic lesions and associated cancers, including mutations in ARID1A, PIK3CA, PTEN, and KRAS, supporting a clonal relationship and malignant progression pathway [39,43]. Recognition of atypical endometriosis at the time of surgery or biopsy should prompt careful histopathologic evaluation and consideration of closer clinical follow-up.

### 5.4. Importance of Clinical Suspicion in Women During Peri-Menopause and Post-Menopause

Perhaps the most critical diagnostic tool is maintaining a high index of clinical suspicion. Clinicians may mistakenly assume that endometriosis “burns out” during peri- and post-menopause; however, persistent or newly symptomatic disease, reactivated by endogenous estrogen production or exogenous sources such as HRT, remains possible [40,41,42]. Key triggers to consider endometriosis include the following:

- Non-cyclical pelvic pain without clear alternative cause;
- Complex adnexal masses in women with a history of endometriosis;
- Gastrointestinal or urinary symptoms suggestive of deep infiltrating disease;
- A history of severe dysmenorrhea or infertility in earlier life, even if previously undiagnosed [45].

Early consideration of endometriosis in the differential diagnosis can lead to timely imaging, appropriate surgical planning, and better outcomes for these patients.

### 5.5. Risk Stratification for Surveillance

Given the potential for malignant transformation in women with longstanding endometriosis, especially those retaining lesions into later life, careful risk assessment is critical [43,44]. Recognized risk factors include the following:

- Long duration of disease, particularly over 10 years;
- Large ovarian endometriomas;
- Older age at diagnosis or presentation, especially post-menopausal women;
- Use of unopposed estrogen therapy, which may stimulate residual endometriotic tissue.

Studies indicate that women with OE present for more than 10 years face an elevated standardized incidence ratio (SIR ~4.2) for ovarian cancer compared to women without endometriosis. The risk is notably higher in older women presenting with multilocular cysts and solid components on imaging [37,42]. Additionally, the estimated lifetime risk of malignant transformation in endometriosis is estimated at approximately 0.3–1.6%, with endometrioid and clear cell carcinoma being the most common histologic subtypes [37,42].

Nezhat et al. discuss the necessity of individualized management strategies for endometriosis, integrating factors such as patient age, reproductive goals, family history, and lesion morphology [44]. Two distinct types of endometriomas have been described: type I, characterized by superficial peritoneal and ovarian involvement, and type II, arising from functional ovarian cysts secondarily invaded by cortical endometriosis. Hormonal therapy generally results in incomplete regression of lesions, whereas ovulation-suppressing regimens may reduce recurrence, particularly in type II cases. Surgical interventions, including unilateral oophorectomy or complete excision of all visible endometriotic tissue, have been associated with a significantly decreased risk of subsequent ovarian cancer [43,44,46,47]. Although post-menopausal women were included in this study, the numbers were limited, precluding definitive conclusions regarding the applicability of these findings to this population [29].

**Figure 1:**
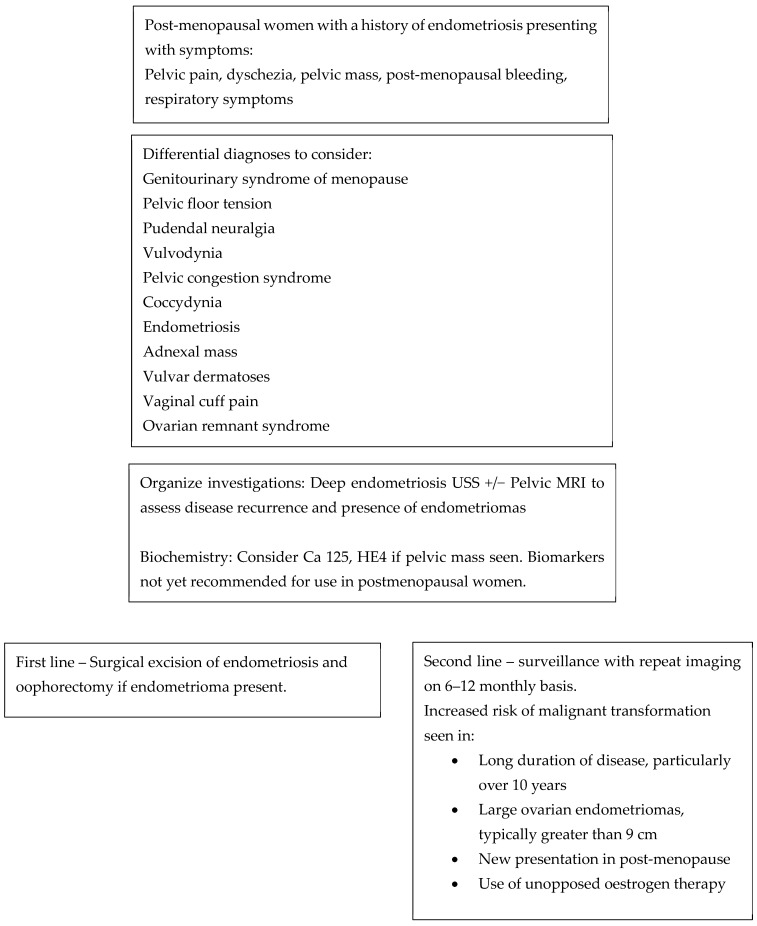
Flow chart to consider when reviewing a menopausal woman with a history of endometriosis.

For patients who decline surgery, surveillance protocols are not clearly defined. Annual TVUS is recommended for known endometriomas due to the ~1% risk of malignant transformation. For previously identified DE, a reasonable strategy is initial follow-up ultrasound or MRI at 6 months has been suggested, then at individualized intervals based on lesion progression or clinical symptoms [48,49]. Surveillance decisions should be tailored to patient goals, imaging findings, and overall health [13,22,43]. Figure 1 illustrates a flowchart to consider when managing a post-menopausal woman with a history of endometriosis.

## 6. Conclusions and Future Directions

While traditionally viewed as a disease of reproductive-age women, endometriosis remains a clinically relevant and often under-recognized condition in the peri- and post-menopausal population. Increasing evidence highlights that endometriotic lesions may persist or even arise de novo after menopause, driven by mechanisms such as local estrogen production, immune dysregulation, progesterone resistance, and epigenetic reprogramming. These factors contribute to sustained inflammation and symptomatology, even in a hypoestrogenic environment.

The clinical presentation in women during peri- and post-menopause is often atypical, with non-cyclical pelvic pain, bowel and bladder symptoms, or pelvic masses frequently misattributed to other age-related pathologies. Diagnostic delays are further compounded by limited epidemiological data, especially in low-resource settings and among underrepresented populations, where cultural taboos and systemic biases may hinder timely recognition and care.

Imaging—ultrasound and MRI—and selected biomarkers play an important role in diagnosis, although neither offers definitive differentiation between benign and malignant lesions. The potential for malignant transformation, especially in cases of long-standing DE, underscores the need for surveillance and risk stratification. In terms of management, surgery remains a cornerstone in peri- and post-menopausal women, particularly when malignancy is suspected. However, individualized approaches—balancing risks, comorbidities, and patient goals—must guide decisions around HRT, non-hormonal options, and the role of surveillance. The role of HRT is a complex one. Considerations must be given not only to the proposed increased risk of reactivation of endometriosis and the patient’s increased cardiovascular risk factors in the context of endometriosis, but also a further higher risk in menopause.

As research evolves, there is an urgent need to develop and refine guidelines specific to the management of endometriosis in peri- and post-menopausal women, incorporating not only clinical and imaging criteria but also considerations around cardiovascular health, bone density, and quality of life. Ensuring equitable access to diagnosis and treatment for all populations remains a critical objective in addressing this long-neglected aspect of endometriosis care.

## Figures and Tables

**Table 1:** HRT that may be considered in post-menopausal women with endometriosis.

Treatment Type	Components/Examples	Use in Women with Endometriosis	Risks/Considerations	Recommendation
Combined HRT	Estrogen + Progestogen	Preferred if uterus is intact	Progestogen opposes estrogenic stimulation of residual lesions	Recommended with caution [26,27,33]
Estrogen-only HRT	Estradiol alone	Contraindicated if uterus intact or history of endometriosis	Risk of stimulating residual or dormant disease	Avoid unless hysterectomy and no endometriosis history [13,33]
Tibolone	Synthetic steroid with mixed hormonal activity	May be considered in inactive disease	Limited data; may reactivate endometriosis in some	Use cautiously [30]
Local (vaginal) estrogen	Vaginal cream, tablet, ring	For urogenital symptoms	Minimal systemic absorption	Generally safe, monitor for symptoms [26]
Progestogen-only therapy	e.g., Norethisterone, LNG-IUS	Suppresses endometriosis	Can be used without estrogen if HRT is not desired	Option in selected cases [34]
Aromatase Inhibitors (AIs)	Letrozole, Anastrozole	Used in recurrent or severe post-menopausal endometriosis	Can cause bone loss, menopausal symptoms; often combined with progestogen or bisphosphonates	Consider for refractory cases under specialist care [28,35]
